# Prediction Method of Soft Fault and Service Life of DC-DC-Converter Circuit Based on Improved Support Vector Machine

**DOI:** 10.3390/e24030402

**Published:** 2022-03-13

**Authors:** Yuntao Hou, Zequan Wu, Xiaohua Cai, Zhongge Dong

**Affiliations:** Heilongjiang Academy of Agricultural Machinery Sciences, Heilongjiang Academy of Agricultural Sciences, Harbin 150081, China; wzq@haas.cn (Z.W.); caixiaohua@haas.cn (X.C.); dongdongshigedanxiaogui@haas.cn (Z.D.)

**Keywords:** DC-DC-converter circuit, soft-fault prediction, service-life estimation, support-vector machine

## Abstract

A data-driven prediction method is proposed to predict the soft fault and estimate the service life of a DC–DC-converter circuit. First, based on adaptive online non-bias least-square support-vector machine (AONBLSSVM) and the double-population particle-swarm optimization (DP-PSO), the prediction model of the soft fault is established. After analyzing the degradation-failure mechanisms of multiple key components and considering the influence of the co-degradation of these components over time on the performance of the circuit, the output ripple voltage is chosen as the fault-characteristic parameter. Finally, relying on historical output ripple voltages, the prediction model is utilized to gradually deduce the predicted values of the fault-characteristic parameter; further, in conjunction with the circuit-failure threshold, the soft fault and the service life of the circuit can be predicted. In the simulation experiment, (1) a time-series prediction is made for the output ripple voltage using the model proposed herein and the online least-square support-vector machine (OLS-SVM). Comparative analyses of fitting-assessment indicators of the predicted and experimental curves confirm that our model is superior to OLS-SVM in both modeling efficiency and prediction accuracy. (2) The effectiveness of the service life prediction method of the circuit is verified.

## 1. Introduction

The electric drive-control system of a seed-metering device serves as the core of the electronic control plot seeder. Its operating performance decides whether the seeding accuracy satisfies the needs [1,2]. As an important part of the secondary power source for the electric drive system of the seed-metering device, the direct-current-direct-current (DC-DC)-converter is important for stable, accurate, and safe seeding. Predicting its faults in advance provides a reference for estimating the service life and avoids impacting on plot-seeding experiments.

System faults consist of hard faults and soft faults. Hard faults mean the system is completely out of action (suddenly); soft faults suggest that the system is gradually losing its function and is finally subject to degradation failure [3]. Along with the continuous improvement of production processes, the system components have a longer service life, and more system faults fall under degradation failures, namely soft faults. Many researchers have made significant contributions to the prediction of soft faults in circuits. For instance, Saha, Patil, and Zhou et al. predicted the faults of electronic devices such as power metal-oxide-semiconductor field-effect transistor (MOSFET), insulated gate bipolar translator (IGBT) performance module, and aluminum electrolytic capacitor, and estimated their service life, respectively [4,5,6]. Ren Lei et al. [7] proposed an online $R_{c}$ (ESR, Equivalent Series Resistance) estimation method for the output capacitor of the Boost converter by analyzing the output ripple voltage. In [8,9,10], S. Dusmez, Li Zhongliang, X. Duan et al. adopted current sensors in order to acquire capacitive current; the average power loss $\bar{P_{c}}$ of the capacitor was calculated based on the measured capacitive voltage and current; by using the equation $R_{c}=\bar{P_{c}}/I_{c}^{2}$, the *R_c_* of the electrolytic capacitor was estimated. Specifically, X. Duan et al. [10] adopted a band-pass filter in order to process the acquired capacitive voltage and current to obtain $R_{c}$ and *C_value_* (capacity of capacitor) of the capacitor within a certain frequency range. However, the use of the filter has led to higher costs and slower parameter-detection rates. Tang et al. [11] established the Buck-converter model based on the hybrid-system theory and identified the capacitor’s characteristic parameters $R_{c}$ and *C_value_* by means of the least-square method. Yet, this method relies on the acquisition of inductive current, output voltage and switch-status signal, and raises requirements for the sampling rate of signals. Lu et al. [12] set up the Boost-converter hybrid-system model using the same method, and the problem of identifying the characteristic parameters of components was transformed into the problem of the global optimization of a multivariable fitness function where $R_{c}$ and *C_value_* were solved through an optimization algorithm. In [13,14], a fault-detection electronics scheme was applied to the insulated gate bipolar transistor (IGBT) by M. A Rodríguez-Blanco and Xinchang Li et al., which was based on online monitoring of the collector current slope signal during the turn-on transient. Sun et al. [15] investigated the application of single-input–single-output (SISO) and multiple-input–single-output (MISO) neural networks for the online monitoring of IGBTs. Moreover, Dusmez et al. [16,17] considered the inductive resistance, the *R_c_* of the electrolytic capacitor, and the drain-source on-resistance of a power MOSFET in the Boost converter and obtained the transfer-function model between the inductive current and the output voltage; the value of the on-resistance $R_{on}$ was then estimated online with the help of software-frequency-response analysis (SFRA). This method applies to circuits under the continuous conduction mode (CCM) and the discontinuous conduction mode (DCM), but it requires the detection of inductive current, and the value of the capacitance $R_{c}$ limits its applicability. Wu et al. [18] utilized the bond-graph theory for modeling the Boost converter in order to yield redundant parsing expressions, and the genetic algorithm was combined in order to identify the drain-source on-resistance $R_{on}$ of a power MOSFET. Sun et al. [19] set up the Boost-converter hybrid-system model based on the hybrid-system theory and capitalized on the particle-swarm-optimization algorithm to identify $R_{on}$, which achieved the simultaneous detection of the characteristic parameters of multiple components in the circuit but required a certain sampling frequency of circuit-detection signals. All the above methods revealed the performance status of a component by detecting the changes in its parameters, thus predicting the faults and service life of the system. Nevertheless, they failed to take an all-sided consideration of how the degradation of other components affects the performance of the DC–DC converter.

To sum up, the current methods used for predicting the faults and service life of the DC–DC converter are plagued by the following issues: (1) they need to detect a wide variety of fault signals and generally have to detect current data, but there are a limited number of detection methods and the detection costs are very high; (2) they mainly focus on the research of characteristic parameters for the faults of a single component, and the degradation-prediction results are outputted based on the changes in the characteristic parameters. In a word, they fail to identify and predict the faults of all the key components and estimate the overall service life of the converter, which limits their applicability to a great extent.

Although the system modeling of the DC–DC-converter circuits can effectively solve the above issues, it is impossible to establish accurate circuit-level degradation models using electronic components such as the power switching tube, diode, and electrolytic capacitor due to their nonlinearity. Consequently, data-driven soft-fault-prediction and service-life-estimation methods were proposed in this study in order to achieve a reliable assessment of the overall performance of the circuit by making full use of the components’ degradation information.

Compared to traditional modeling based on Kirchhoff’s voltage and current laws, a parameter-identification method that uses data-driven models avoids the derivation of complex circuit equations. Specifically, relying on the feature extraction of a system’s historical data, this method can predict its future status based on current information, judging whether a fault will occur and estimating its service life. Data-driven methods are mainly categorized into mathematical statistics and machine-learning methods, such as the support-vector machine [20,21,22,23], Kalman filtering [24,25], Gaussian process regression [26,27,28], the neural network [29,30,31,32,33], the particle filter [34], the evidence theory [35], grey prediction [36,37], Markov [38,39], and the Bayesian network [40,41]. Only a signal analysis of the measured data is required for these methods to facilitate modeling and prediction without the need to establish complex physical or mathematical models involving massive computation. However, their weaknesses are also obvious: (1) the prediction accuracy of some algorithms is greatly hinged on technological parameters, such as the setting of the learning rate and the number of hidden layers for the neural network, and the configuration of penalty and breadth factors for the support-vector machine (SVM), whose prediction accuracy will be greatly affected if the parameters are not properly configured; (2) the other algorithms are characterized by high complexity and massive computation, resulting in low modeling efficiency. For example, the Gaussian process-regression method can only be used for predicting small data samples due to massive computations. On the other hand, the particle-filter algorithm functions well in the nonlinear, non-Gaussian system, but it requires large data samples to ensure the probability density of the approximation system, and the system complexity also increases significantly along with the increasing sample-set size.

The least-squares support-vector machine (LSSVM), a variant of the standard SVM, was developed by Suykens and Vandewalle [42,43,44]. The LSSVM introduces the least-squares linear system as a loss function, and has better anti-noise ability and faster operation speed than the standard SVM. In the present work, the LSSVM is improved in order to perform the regression prediction of the fault-characteristic parameter (output ripple voltage) of DC–DC converter circuit.

By optimizing the structural-risk forms of the LSSVM and integrating the online-learning method of square-root decomposition, the online non-bias least-square support-vector machine (ONBLSSVM) is proposed to construct the AONBLSSVM model in combination with the adaptive deterministic algorithm of sliding-time-window length, which can make full use of the features of historical training results and the augmented kernel matrix, and improve the modeling efficiency. Furthermore, double-population particle-swarm optimization (DP-PSO) is applied to the optimized calculation in order to choose the most appropriate model parameters and increase the prediction accuracy. Based on historical data of the output ripple voltage, the fault trend is predicted by means of gradually recursive predicted values until the value reaches the preset failure threshold, thereby achieving the prediction of the soft fault and service life of the circuit.

The rest of the paper is organized as follows. Section 2 is the very core of the paper: in Section 2.1, we introduce the construction of the non-biased form of the LSSVM in detail and discuss the property of the augmented kernel matrix; in Section 2.2, the online sample-addition-and-removal algorithm is deduced based on square-root decomposition; in Section 2.3, the adaptive deterministic algorithm of the sliding-time-window length is proposed; in Section 2.4, the DP-PSO is deduced for the optimized computation of hyper-parameters in the prediction model. Section 3 introduces the establishment of degradation models for key components, the selection of characteristic parameters for circuit-level faults, the establishment of the prediction model, simulation experiments and result analyses. Finally, we conclude our work in Section 4.

## 2. Fault Prediction Model

### 2.1. Model Initialization

The initial parameter sample sets within the sliding-time window were adopted for constructing a model at the initial moment. Supposing that the length of the sliding-time window is defined as l, the training sample set at the initial moment can be expressed as $\left(x_{i},y_{i}\right)\left(i=1,2,3\ldots l\right)$, where in the model inputs $x_{i}\in R^{n}$, and the model outputs $y_{i}\in R$. By optimizing the structural-risk forms of LSSVM [45,46,47,48] and adding the item $b^{2}/2\lambda^{2}$ (λ > 0), the objective function and constraint condition of the prediction model can be expressed as:

(1) $$\begin{array}{c} \begin{array}{l} \min\frac{1}{2}\left(\omega \cdot \omega \right)+\frac{1}{2\lambda^{2}}b^{2}+\frac{1}{2}C\sum_{i=1}^{l}\xi_{i}^{2}\\ s.t.y_{i}-\omega^{T}\varphi \left(x_{i}\right)-b=\xi_{i} \end{array}\}\\ i=1,2,\ldots ,l \end{array}$$

where ω is the normal vector, which determines the direction of the hyperplane; (⋅) is an inner product operation; $\varphi \left(x_{i}\right)$ represents the eigenvector after mapping $x_{i}$; λ is an introduced parameter; b is the bias term of the LSSVM, which determines the distance between the hyperplane and the origin. ξ is a relaxation variable to avoid over-complexity of the model and to improve the generalization performance of the model; *C* is the penalty parameter, and a larger *C* corresponds to a smaller tolerance of the objective function to the fitting error.

Supposing that $\omega^{\prime }=\left(\omega ,b/\lambda \right)$, Equation (1) is transformed into:

(2) $$\begin{array}{c} \begin{array}{l} \min\frac{1}{2}\left(\omega^{\prime }\cdot \omega^{\prime }\right)+\frac{1}{2}C\sum_{i=1}^{l}\xi_{i}^{2}\\ s.t.y_{i}-\omega^{\prime T}\left(\varphi \left(x_{i}\right),\lambda \right)=\xi_{i} \end{array}\}\\ i=1,2,\ldots ,l \end{array}$$

By establishing the Lagrange function (Equation (3)) and integrating *KKT* conditions (Karush-Kuhn-Tucker conditions), the function optimization under the constraint condition can eliminate the constraint condition, namely:

(3) $$L=\frac{1}{2}\left(\omega^{\prime }\cdot \omega^{\prime }\right)+\frac{1}{2}C\sum_{i=1}^{l}\xi_{i}^{2}-\sum_{i=1}^{l}\alpha_{i}\left[\omega^{\prime T}\left(\varphi \left(x_{i}\right),\lambda \right)+\xi_{i}-y_{i}\right]$$

where in $\alpha_{i}$ is the Lagrange multiplier.

By taking the derivatives of $\omega^{\prime },\;\xi_{i},\;\mathrm{and}\;\alpha_{i}$, respectively, the following equations are obtained:

(4) $$\{\begin{array}{l} \frac{\partial L}{\partial \omega^{\prime }}=0\to \omega^{\prime }=\sum_{i=1}^{l}\alpha_{i}\left(\varphi \left(x_{i}\right),\lambda \right)\\ \frac{\partial L}{\partial \xi_{i}}=0\to \alpha_{i}=C\xi_{i}\\ \frac{\partial L}{\partial \alpha_{i}}=0\to \omega^{\prime T}\left(\varphi \left(x_{i}\right),\lambda \right)+\xi_{i}-y_{i}=0 \end{array}$$

For i=1,2,…,l, $\omega^{\prime }\;\mathrm{and}\;\xi_{i}$ are eliminated, so Equation (4) can be transformed into:

(5) $$\left(K+\lambda^{2}E+C^{-1}I\right)\alpha =Y$$

where in *E* is an l×l all-ones matrix; *I* is an l×l unit matrix; $K_{i,j}=\left(\varphi \left(x_{i}\right)\cdot \varphi \left(x_{j}\right)\right)=k\left(x_{i},x_{j}\right);Y=\left(y_{1},y_{2},\ldots ,y_{l}\right);\alpha ={\left(\alpha_{1},\alpha_{2},\ldots ,\alpha_{l}\right)}^{T}$.

The initial prediction model is mathematically transformed into:

(6) $$f\left(x\right)=\sum_{i=1}^{l}\alpha_{i}\left(k\left(x,x_{i}\right)+\lambda^{2}\right)$$

It can be seen from Equation (6) that by introducing the parameter λ, the mathematical model of the LSSVM is optimized, and the goal of eliminating the bias term of the regression function is achieved.

Supposing that $H=K+\lambda^{2}E+C^{-1}I\left(\lambda >0,C>0\right)$, Equation (5) can be simplified into Hα=Y(*H* is the augmented kernel matrix). Thus, it is verified that *H* is not only a symmetric matrix but also a positive definite matrix, so it can be decomposed through the square-root method. *H* can be solely decomposed into $H=U^{T}U$, wherein U is the upper triangular matrix. Matrix elements $u_{ii}$, $u_{ij}$ in U can be determined by the following equation:

(7) uii=(hii−∑k=1i−1uki)12,i=1,2,…,luij=(hij−∑k=1i−1ukiukj)/uii,j>i

Supposing that P=Uα, $U^{T}P=Y$, the Lagrange-multiplier vector α in Equation (5) can be computed by using the following equation:

(8) $$\begin{array}{c} p_{i}=\left(y_{i}-\sum_{k=1}^{i-1}u_{ki}p_{k}\right)/u_{ii}\\ a_{i}=\left(p_{i}-\sum_{k=i+1}^{n}u_{ik}x_{k}\right)/u_{ii} \end{array}$$

where $p_{i}$ is the *i-*th component of *P*, and $\alpha_{i}$ is the *i-*th component of α.

The optimized model offers a simpler solving method than the LSSVM does.

### 2.2. Online Model Updates

As the sliding-time window moves within the sample set, it will surely lead to dynamic updates of the training sample sets stored in the time window (such as adding new samples or removing old samples). How to dynamically update the prediction model at minimum computation costs while satisfying the requirements for prediction accuracy and modeling speed remains an issue to be tackled.

(1) Adding samples

Supposing that *l* samples [49] have been stored in the sliding-time window at time *t,* the training set is expressed as $\left\{\left(x_{i},y_{i}\right)\right\}\left(i=t+1,t+2,\ldots ,t+l\right)$. Along with the translation of the time window, a new sample $\left(x_{t+l+1},y_{t+l+1}\right)$ shall be added.

In the ONBLSSVM algorithm, the Lagrange-multiplier vector α, the output set Y of the samples within the sliding-time window, and the kernel-function matrix K are all mathematical models about time *t*, as shown below:

(9) $$\alpha \left(t\right)={\left(\alpha_{t+1},\alpha_{t+2},\ldots ,\alpha_{t+l}\right)}^{T}$$

(10) $$Y\left(t\right)={\left(Y_{t+1},Y_{t+2},\ldots ,Y_{t+l}\right)}^{T}$$

(11) $$K_{i,j}\left(t\right)=k\left(x_{i},x_{j}\right)$$

Supposing $H\left(t\right)=K\left(t\right)+\lambda^{2}E+C^{-1}I\;$(the determination method of λ and C is detailed in Section 2.4), α(t) can be solved through H(t)α(t)=Y(t). The output of the online non-bias least-square support-vector machine (ONBLSSVM) is written as:

(12) $$f\left(x_{t+l+1}\right)=\sum_{i=t+1}^{t+l}\alpha_{i}\left(k\left(x_{t+l+1},x_{i}\right)+\lambda^{2}\right)$$

Due to the positive symmetry of H(t), supposing that $H\left(t\right)=U{\left(t\right)}^{T}U\left(t\right)$, the matrix K(t) is an l×l order matrix at time t.

(13) $$K\left(t\right)=\left[\begin{array}{ccc} k\left(x_{t-l+1},x_{t-l+1}\right) & \cdots & k\left(x_{t-l+1},x_{t}\right)\\ \vdots & \ddots & \vdots \\ k\left(x_{t},x_{t-l+1}\right) & \cdots & k\left(x_{t},x_{t}\right) \end{array}\right]$$

Correspondingly

(14) $$H\left(t\right)=\left[\begin{array}{ccc} k\left(x_{t-l+1},x_{t-l+1}\right)+\lambda^{2}+\frac{1}{C} & \cdots & k\left(x_{t-l+1},x_{t}\right)+\lambda^{2}\\ \vdots & \ddots & \vdots \\ k\left(x_{t},x_{t-l+1}\right)+\lambda^{2} & \cdots & k\left(x_{t},x_{t}\right)+\lambda^{2}+\frac{1}{C} \end{array}\right]$$

It can be known from the learning results at time *t* that $H\left(t\right)=U{\left(t\right)}^{T}U\left(t\right)$, and a new sample $\left(x_{t+l+1},y_{t+l+1}\right)$ is added at time *t* + 1, so the following equation can be obtained:

(15) $$H\left(t+1\right)=\left[\begin{array}{cc} H\left(t\right) & V\left(t+1\right)\\ V{\left(t+1\right)}^{T} & v\left(t+1\right) \end{array}\right]\in R^{\left(l+1\right)\times \left(l+1\right)}$$

where in $V\left(t+1\right)={\left[k\left(x_{t+l+1},x_{t+1}\right)+\lambda^{2},\ldots ,k\left(x_{t+l+1},x_{t+l}\right)+\lambda^{2}\right]}^{T}$; $v\left(t+1\right)=k\left(x_{t+l+1},x_{t+l+1}\right)+\lambda^{2}+C^{-1}$.

Now, U(t+1) is solved so that $H\left(t+1\right)=U{\left(t+1\right)}^{T}U\left(t+1\right)$. As H(t+1) is a symmetric positive matrix, the square-root method is adopted for solving H(t+1):

(16) $$U\left(t+1\right)=\left[\begin{array}{cc} U\left(t\right) & W\left(t+1\right)\\ 0^{T} & w\left(t+1\right) \end{array}\right]$$

where in W(t+1) and w(t+1) are the *l* dimensional column vector and the real number, respectively.

Besides, as $H\left(t+1\right)=U{\left(t+1\right)}^{T}U\left(t+1\right)$ and Equation (16), in the calculation of the matrix H(t+1) which is obtained after the addition of a new sample $\left(x_{t+l+1},x_{t+l+1}\right)$ at time *t* + 1, the previous calculation result U(t) can be used to improve the computation efficiency.

(2) Removing samples

Supposing that the new sample $\left(x_{t+l+1},x_{t+l+1}\right)$ is added and the old sample $\left(x_{t+1},x_{t+1}\right)$ is removed from the training sample set, the solving matrix $\overset{\wedge }{H}\left(t+1\right)$ of the Lagrange multiplier is obtained. By repartitioning H(t+1) and U(t+1), the following equations can be obtained:

(17) $$H\left(t+1\right)=\left[\begin{array}{cc} \hat{v}\left(t-l+1\right) & {\hat{V}}^{T}\left(t+1\right)\\ \hat{V}\left(t+1\right) & \hat{H}\left(t+1\right) \end{array}\right]$$

where the matrix $\overset{\wedge }{H}\left(t+1\right)$ is an l×l order matrix; $\overset{\wedge }{V}\left(t+1\right)$ and $\overset{\wedge }{v}\left(t-l+1\right)$ are the *l* dimensional column vector and the real number, respectively.

(18) $$U\left(t+1\right)=\left[\begin{array}{cc} \hat{w}\left(t-l+1\right) & {\hat{W}}^{T}\left(t+1\right)\\ 0 & \hat{U}\left(t+1\right) \end{array}\right]$$

where the matrix $\overset{\wedge }{U}\left(t+1\right)$ is an l×l order matrix; $\overset{\wedge }{W}\left(t+1\right)$ and $\overset{\wedge }{w}\left(t-l+1\right)$ are the *l* dimensional column vector and the real number, respectively.

It can be seen from $H\left(t+1\right)=U{\left(t+1\right)}^{T}U\left(t+1\right)$ that:

(19) $$\hat{H}\left(t+1\right)={\hat{U}}^{T}{\left(t+1\right)}^{T}\hat{U}\left(t+1\right)+{\hat{W}}^{T}{\left(t+1\right)}^{T}{\hat{W}}^{T}\left(t+1\right)$$

According to Equation (19), the new Lagrange-multiplier vector can be solved, thus yielding the prediction model at time t+1.

### 2.3. Adaptive Selection of the Sliding-Time-Window Length

To establish the AONBLSSVM prediction model, the length of the sliding-time window for storing training data shall be determined first. If the time window is too short, fewer data will be stored, possibly leading to the consequences that the samples are not representative enough and the model’s prediction accuracy is not satisfactory; if it is too long, overfitting may occur, and the online modeling speed will be reduced [50,51]. As a result, an algorithm for adaptively selecting the length of the sliding-time window shall be designed based on data features and preset prediction accuracy.

Supposing that there is a sample set $W=\left\{s_{1},s_{2}\right\}$ within the initial sliding-time window; θ is defined as the prediction-error threshold of the sample and ε refers to the relative-decrement threshold of the objective function. During the adjustment of the window length, the latest samples are continuously added in order to dynamically update the model, and the predicted value of the next sample is offered based on the updated model. The computation may terminate in order to output the length of the sliding-time window if the following two conditions are met: (1) the time-series-prediction error of the training set is less than θ; (2) the relative decrement $\Delta_{t-1}$ of the objective function is less than the threshold ε for n continuous times.

The calculation equation of the objective function $Q_{t-1}$ is written as:

(20) $$\begin{array}{lll} Q_{t-1} & = & \frac{1}{2}\left(\omega_{t-1}^{\prime }\cdot \omega_{t-1}^{\prime }\right)+\frac{1}{2}C\sum_{i=1}^{t-1}\xi_{i}^{2}\\ & = & \frac{1}{2}\sum_{i=1}^{t-1}\sum_{i=1}^{t-1}\left[\alpha_{i}\alpha_{j}\left(k\left(x_{i},x_{j}\right)+\lambda^{2}\right)\right]+\\ & & \frac{1}{2}C\sum_{i=1}^{t-1}{\left[y_{i}-\sum_{j=1}^{t-1}\alpha_{j}\left(k\left(x_{i},x_{j}\right)+\lambda^{2}\right)\right]}^{2} \end{array}$$

Supposing that $Q_{t-2}=Q_{t-1}/l$, the relative decrement of the objective function $\Delta_{t-1}$ is expressed as:

(21) $$\Delta_{t-1}=\frac{\left|Q_{t-1}-Q_{t-2}\right|}{Q_{t-1}}$$

**Figure 1:**
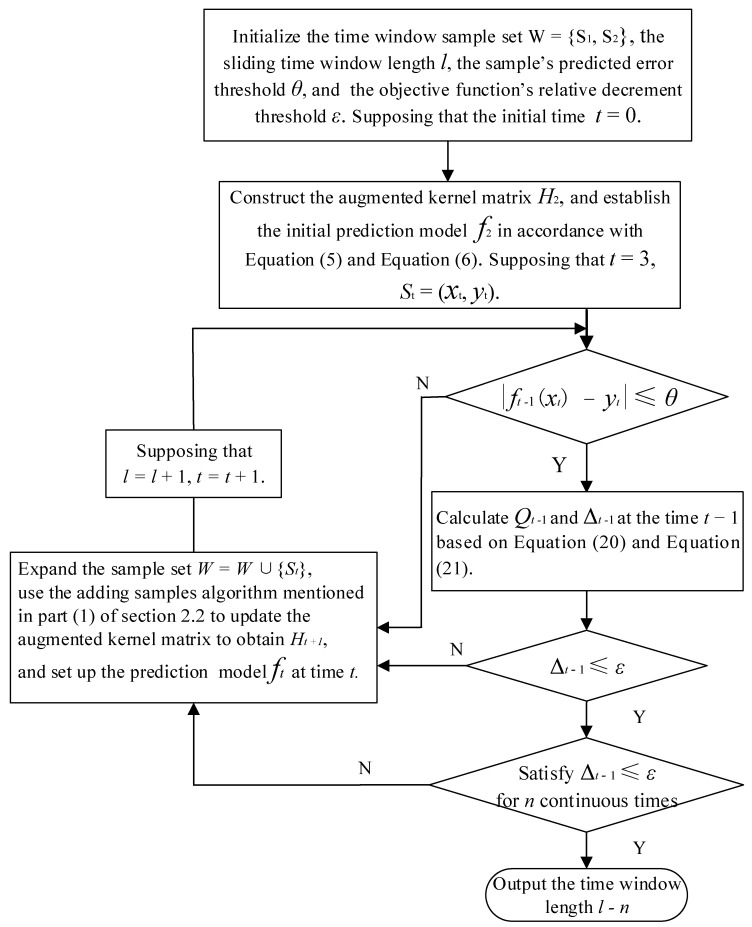
Algorithm flowchart for adaptively selecting the length of the sliding-time window.

The major operating steps of the algorithm for adaptively selecting the length of the sliding-time window are shown in Figure 1:

After finalizing the length of the sliding-time window, as the time window continues to move among samples, the online modeling of AONBLSSVM is completed through the dynamic addition and removal of samples.

### 2.4. Optimized Computation of Model Parameters Based on DP-PSO

The AONBLSSVM model parameters that require optimized computation include the penalty factor *C*, the introduced parameter λ, and the kernel function’s breadth factor $\sigma^{2}$ (the Gaussian kernel function is adopted for the model). During the modeling process (based on given samples), it is a top priority to obtain combined optimal solutions of model parameters for modeling [52].

Particle-swarm optimization (PSO) works well in function optimization [53,54], but it is easily trapped at extreme points on the local scale [55] and its convergence rate at the later period is quite slow [56,57]. To make up for the defects of PSO, the concept of population co-evolution was introduced into PSO in this study [58,59,60], and online dynamic adjustment of the acceleration factor [61] was adopted for the tracking of current search results and the online real-time rectification of search strategies.

The specific method is shown as follows: the particle swarm s is partitioned into two sub-swarms $Q_{1}$ and $Q_{2}$. $Q_{1}$ contains $s_{1}$ particles, while $Q_{2}$ consists of $s_{2}$ particles; $s=s_{1}+s_{2}$. $Q_{1}$ adopts the rapidly convergent evolution equation for fast and optimized convergence within a small range between the optimal global position and the optimal individual position; $Q_{2}$ adopts the evolution equation with global searching ability. When a new optimal global position is searched, $Q_{1}$ is guided to reach the new optimal position for local searching through information exchange between individuals.

Specific evolution equations are shown below:

(22) $$\begin{array}{ll} Q_{1}:v_{ij}{}^{1}\left(t+1\right)= & w^{1}\times v_{ij}{}^{1}\left(t\right)+c_{1}\times rand\left(\right)\times \left(p^{1}{}_{ij}\left(t\right)-x_{ij}{}^{1}\left(t\right)\right)\\ & +c_{2}\times rand\left(\right)\times \left(p_{gj}{}^{1}\left(t\right)-x_{ij}{}^{1}\left(t\right)\right) \end{array}$$

where $v_{ij}^{1}\left(t+1\right)$ is the velocity of the particle at time *t* + 1; $p_{ij}^{1}\left(t\right)$ is the optimal historical position of the particle at time *t;* $p_{gj}^{1}\left(t\right)$ is the historical optimal position of the population $Q_{1}$; $x_{ij}^{1}\left(t\right)$ and $v_{ij}^{1}\left(t\right)$ are the position and velocity of the particle at time *t*; the inertia weight $w^{1}=0.3$; $c_{1}$ and $c_{2}$ are the acceleration factors; and rand() is a random number within the range of [0,1].

(23) $$\begin{array}{c} \begin{array}{c} Q_{2}:v_{ij}\left(t+1\right)=w\left(t\right)\times v_{ij}\left(t\right)+c_{1}\times r_{1j}\left(t\right)\times \left(p_{ij}\left(t\right)-x_{ij}\left(t\right)\right)\\ +c_{2}\times r_{2j}\left(t\right)\times \left(p_{gj}\left(t\right)-x_{ij}\left(t\right)\right) \end{array}\\ w\left(t\right)=0.9-\frac{t}{T_{\max}}\times 0.5 \end{array}$$

where $v_{ij}^{2}\left(t+1\right)$ is the velocity of the particle at time *t* + 1; $p_{ij}^{2}\left(t\right)$ is the optimal historical position of the particle at time *t;* $p_{gj}^{2}\left(t\right)$ is the historical optimal position of the population $Q_{2}$; $x_{ij}^{2}\left(t\right)$ and $v_{ij}^{2}\left(t\right)$ are the position and velocity of the particle at time *t*; the inertia weight $w^{2}\left(t\right)$ is the inertia weight; $c_{1}$ and $c_{2}$ are the acceleration factors; and $r_{1j}\left(t\right)$ and $r_{2j}\left(t\right)$ are random numbers within the range of [0,1].

Acceleration factors $c_{1}\;\mathrm{and}\;c_{2}$ of dynamic adjustment Equations (22) and (23) in the arc-tangent function are adopted in order to adjust the search strategy in a real-time manner. The equations for $c_{1}\;\mathrm{and}\;c_{2}$ are written as:

(24) $$\begin{array}{ll} c_{1}\left(t\right)= & c_{1start}-\left(c_{1start}-c_{1end}\right)\times \\ & \left(\arctan\left(20\times t/T_{\max}-e\right)+\arctan\left(e\right)\right)/l^{\prime } \end{array}$$

(25) $$\begin{array}{ll} c_{2}\left(t\right)= & c_{2start}-\left(c_{2start}-c_{2end}\right)\times \\ & \left(\arctan\left(20\times t/T_{\max}-e\right)+\arctan\left(e\right)\right)/l^{\prime } \end{array}$$

where in $c_{1start}\;\mathrm{and}\;c_{2start}$ are the initial values of $c_{1}\;\mathrm{and}\;c_{2}$, respectively; $c_{1end}\;\mathrm{and}\;c_{2end}$ are final values of $c_{1}\;\mathrm{and}\;c_{2},\;\mathrm{respectively};\;T_{\max}$ is the maximum evolution algebra; e is the adjustment factor; $l^{\prime }=arctan\left(20-e\right)$ + arctane.

**Figure 2:**
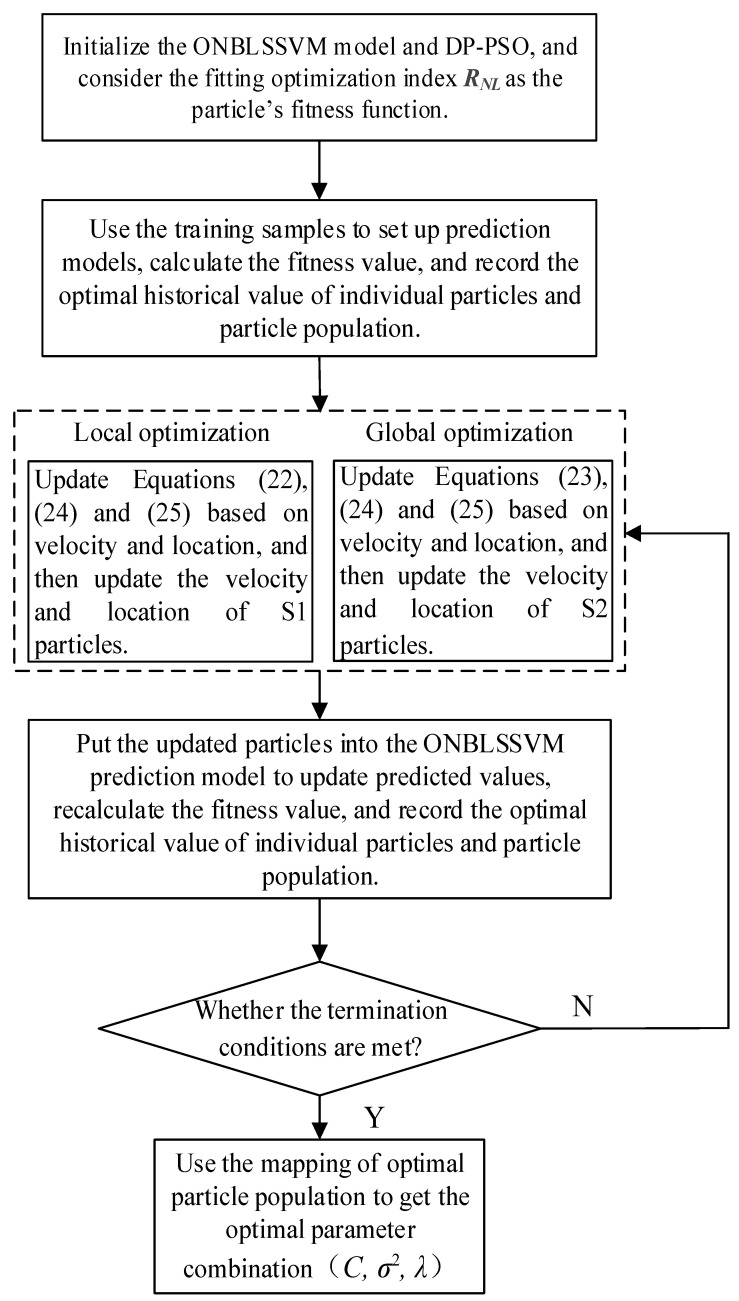
Flowchart of model-parameter optimization based on DP-PSO.

The process of optimizing the model parameters is shown in Figure 2, and the optimization shall terminate when the following conditions are met: (1) the fitting-optimization index [62] $R_{NL}=1-\sqrt{\sum {\left(y_{i}-\overset{\wedge }{y_{i}}\right)}^{2}/\sum y_{i}^{2}}$ between the predicted and target values satisfies the preset error, where $y_{i}$ is real value and $\overset{\wedge }{y_{i}}$ is predicted value; (2) the preset $T_{\max}$ is achieved.

## 3. Simulation Experiments and Result Analyses

### 3.1. Establishment of Degradation Models for Key Components

**Figure 3:**
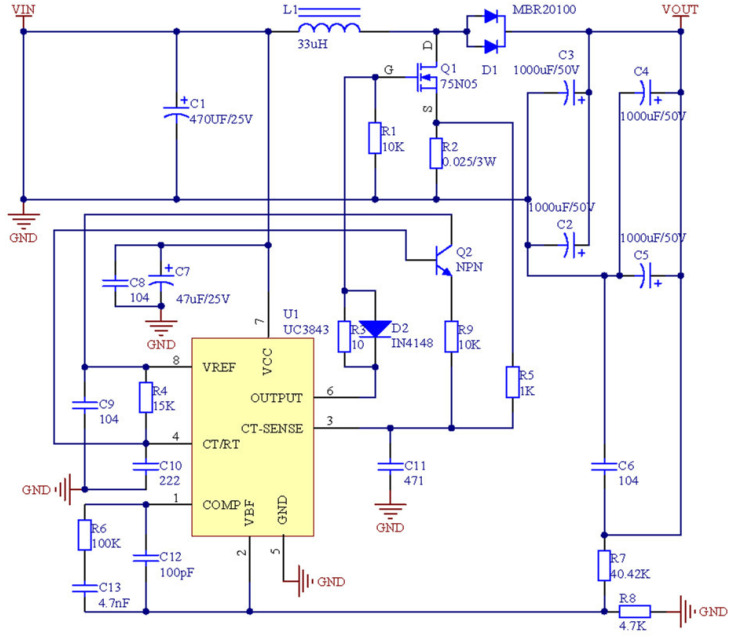
The schematic diagram of DC–DC-converter circuit.

The DC–DC-converter circuit designed in this study is a Boost circuit. As shown below Figure 3, the circuit achieves an input voltage of 12 Vdc, an output voltage of 24 Vdc, an $\mathrm{output}\;\mathrm{ripple}\;\mathrm{voltage}\;\left(V_{out}\left(\max\right)-V_{out}\left(\min\right)\right)\leq 0.1V_{out}$, and an output power $P_{out}=200\;W\left(\mathrm{MAX}\right)$.

By analyzing the failure mechanisms of key components such as the electrolytic capacitor, power MOSFET, diode, and electrical inductor, the performance-degradation models for various components were established to configure the changes in the parameters of components during the circuit-degradation process. On this basis, a circuit-level simulation and performance-degradation analysis were carried out, thus achieving fault prediction and service-life estimation of power-converter circuits.

Performance-degradation models of key components can be obtained from the following equations:

(1) Performance-Degradation Model of Electrolytic Capacitor

Capacitors in real life are all found with the equivalent-series resistance (ESR), among which the ESR for electrolytic capacitors is the largest. Its degradation model is described as [63,64]: 


(26) $${ESR}^{-1}={ESR(0)}^{-1}\cdot (1-k_{ESR}\cdot t_{ESR}\cdot e^{-\frac{4700}{\left(273+T_{ESR}\right)}})$$

This model reveals the mathematical relationship between ESR(t) and its initial value ESR(0), where $T_{ESR}$ represents the kernel temperature, $t_{ESR}$ refers to the working time, and $k_{ESR}$ is a parameter that is only related to the capacitive material.

The wastage of electrolytes increases over time. The performance-degradation model [65], i.e., $\Delta C_{value}\left(t_{c}\right)=\frac{C_{value}\left(0\right)-C_{value}\left(t_{c}\right)}{C_{value}\left(0\right)}\%$ of *C_value_* (capacity of capacitor), is expressed as:

(27) $$\Delta C_{value}\left(t_{C}\right)=0.01\left(e^{\alpha_{1}t_{C}}-\beta_{1}\right)$$

where $t_{c}$ refers to the working time and $\alpha_{1}$ and $\beta_{1}$ are degradation parameters of the model.

The failure condition of the electrolytic capacitor is set as follows [66]: $ESR\left(t_{ESR}\right)\geq 3\times ESR\left(0\right);\Delta C_{value}\;\left(t_{c}\right)\geq 20\%\times C\left(0\right)$.

By referring to the component manual, it can be known that ESR(0)=0.02 Ω in the working environment of $T_{ESR}=27\;℃$. Supposing that $ESR\left(t_{ESR}\right)=3\times ESR\left(0\right),\;t_{ESR}=1500\;h$, and ESR(1500)=0.06 Ω, it can be inferred from Equation (26) that $k_{ESR}=2839$. Therefore, the degradation model of *ESR* over time is established as follows:

(28) $$ESR\left(t_{ESR}\right)=\frac{ESR\left(0\right)}{1-k_{ESR}\cdot t_{ESR}\cdot \exp\left(\frac{-4700}{T_{ESR}+273}\right)}=\frac{0.02}{1-0.000444\cdot t_{ESR}}$$

where in $C_{value}\left(0\right)=1000uF$. Supposing that $\Delta C_{value}\left(t_{c}\right)=20\%,t_{c}=1500\;h$, and the parameter $\beta_{1}\;$= 1, it can be known from Equation (27) that $\alpha_{1}=0.002030$. Then, the degradation model of *C_value_* over time is expressed as:

(29) $$\begin{array}{ll} C_{value} & \left(t_{C}\right)=C_{value}\left(0\right)\cdot \left[1-\Delta C_{value}\left(t_{C}\right)\right]\\ & =1000\times 10^{-6}\left[1-0.01\times \left(e^{0.002030t_{c}}-1\right)\right] \end{array}$$

(2) Performance-Degradation Model of Power MOSFET

On-resistance $R_{on}$ is a key parameter that determines the dissipated power of the MOSFET, whose empirical degradation model is written as:

(30) $$\Delta R_{on}\left(t_{MOS}\right)=\alpha_{2}\left(e^{b_{2}t_{MOS}}-1\right)$$

where in $t_{MOS}$ refers to the MOSFET’s working time; $a_{2}$ and $b_{2}$ are degradation parameters of the model. When $R_{on}>0.045\;\Omega$, it is believed that the MOSFET is out of work [67].

By referring to the component manual, it can be known that 75N05 has a $R_{on}\left(0\right)=0.02\;\Omega$, so it is deemed that the MOSFET is out of work when $R_{on}$ increases to 0.065 Ω. Supposing that $R_{on}=0.045\;\Omega ,\;t_{MOS}=1500\;h$*,* and the model parameter $a_{2}=0.003$ it can be deduced from Equation (30) that the parameter $b_{2}=0.00185$. Therefore, $R_{on}$ is expressed as:

(31) $$\begin{array}{ll} R_{on} & \left(t_{MOS}\right)=R_{on}\left(0\right)+\Delta R_{on}\\ & =0.02+0.003\left(e^{0.00185t_{MOS}}-1\right) \end{array}$$

(3) Performance-Degradation Model of Inductor

During the working process of the inductor, the inductance gradually decreases along with the increase in temperature, making it impossible for the circuit to function normally. The performance-degradation model [68] of the inductor used in this circuit is described as:

(32) $$L\left(t_{L}\right)=L\left(0\right)-\alpha_{3}t_{L}$$

where $t_{L}$, $\alpha_{3}$ and L(0) represent the duration, the performance-degradation parameter, and the initial nominal value, respectively.

Previous experience suggests that the inductor is out of work when $L\left(t_{L}\right)<0.8\times L\left(0\right)$ [69]. Supposing that $L\left(t_{L}\right)=0.8L\left(0\right),t_{L}=1500\;h$, it can be deduced from Equation (32) that $\alpha_{3}=0.0044$. Therefore, *L* value at time $t_{L}$ is expressed as:

(33) $$L\left(t_{L}\right)=L\left(0\right)-0.0044t_{L}$$

(4) Performance-Degradation Model of Power Diode

By referring to the MOSFET, the on-resistance $R_{D}$ can be employed as a characteristic parameter to judge whether a power diode functions normally. Besides, it is believed that the power diode is out of work when $R_{D}$ is greater than the initial value 0.045 Ω [70,71]. The degradation model [72,73] of $\Delta R_{D}$ can be described as:

(34) $$\Delta R_{D}\left(t_{D}\right)=\alpha_{4}\cdot \left(e^{b_{4}t_{D}}-1\right)$$

where $t_{D}$ is the working time of the power diode; $\alpha_{4}$ and $b_{4}$ are degradation parameters of the model.

Supposing that $R_{D}$ has an initial value of $R_{D}\left(0\right)=0.01\;\Omega$ with reference to the component manual, it is believed that the power diode is out of work when $R_{D}$ increases to 0.055 Ω. Supposing that it takes 1500 h for the on-resistance to increase to 0.055 Ω, and that $\alpha_{4}=0.00025,$ it can be known from Equation (34) that $b_{4}=0.0035$. Therefore, the on-resistance $R_{D}$ at time $t_{D}$ is expressed as:

(35) $$\begin{array}{ll} R_{D}\left(t_{D}\right) & =R_{D}\left(0\right)+\Delta R_{D}\left(t_{D}\right)\\ & =0.01+0.00025\left(e^{0.0035t_{D}}-1\right) \end{array}$$

### 3.2. Selection of Characteristic Parameters for Circuit-Level Faults

The simulation circuit of the DC–DC converter was built in the simulation software saber, with an input voltage of 12 Vdc and an output voltage of 24 Vdc. The simulation time was set as 30 ms, with a simulation-step size of 1 us. When the circuit output reached a stable state, the output voltage $V_{out}$ was sampled, and the simulation waveform was drawn.

**Figure 4:**
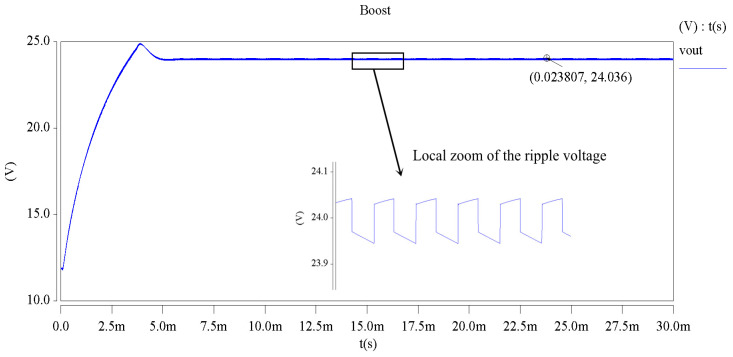
The simulation waveform of output voltage.

It can be known by observing the $V_{out}$ waveform in Figure 4 that the waveform of the output voltage tends to be stable when the simulation experiment is conducted for 5 ms; the output voltage $V_{out}$ fluctuates around 24 V because the DC–DC converter switches between charging and discharging modes during the working process. Consequently, its output-voltage waveform does not exhibit stable DC voltage but is found to be fluctuating, suggesting the presence of ripple voltage $U_{PP}$.

Based on the performance-degradation models of various components from Equation (26) to Equation (35), different values were set for the parameters of each component at a time interval of Δt(Δt=100 h) in sequence since t=0, which were then inputted into the DC–DC simulation circuits of saber for the simulation experiments. It can be known from the simulation analysis that as the working time increases, the waveform of the output ripple voltage $U_{PP}$ always tends to expand over time when the performance of multiple key components C2−C5,L1,MBR20100 and 75N75 degrade at the same time, and the changes are quite noticeable, as detailed in Table 1. Therefore, the output ripple voltage $U_{PP}$ was chosen in this study as a characteristic parameter for the faults of the DC–DC-converter circuits. According to the performance indicators, if $U_{PP}>0.24\;V$, then the circuit is trapped in a fault. The ripple voltage can be obtained by using the equation $U_{PP}=V_{out}\left(\max\right)-V_{out}\left(\min\right)$.

Different values were set for the parameters of each component every 1 h in sequence since t=0, which were then inputted into the DC–DC simulation circuits of the saber to retain the output voltage $V_{out}$ within the stable band (10–30 ms). A total of 1400 groups of ripple voltages $U_{PP}$ from 1–1400 h were obtained using the equation $U_{PP}=V_{out}\left(\max\right)-V_{out}\left(\min\right)$, which formed the characteristic-parameter sample sets of the soft fault of the circuit.

### 3.3. Determination of Parameters for the Prediction Model

DP-PSO was adopted for the optimized computation of the prediction-model parameters, including the penalty factor C, the introduced parameter λ, and the kernel breadth factor $\sigma^{2}$. Three hundred samples were selected, which were set with the following parameters: the swarm quantity S=100, the sub-swarm quantity $s_{1}=35$ and $s_{2}=65$, the maximum evolution algebra $T_{max}=200$ the acceleration factor $c_{1start\;}=2.75,\;c_{1end\;}=1.25,\;c_{2start}=0.5\;\mathrm{and}\;c_{2end}=2.25$. The penalty factor *C* varied within $\left[10^{-2},\;10^{3}\right]$ the Gaussian kernel function breadth factor $\sigma^{2}$ varied within $\left[10^{-2},\;10^{2}\right]$ and the parameter λ varied within $\left[10^{-3},\;10\right].$

**Figure 5:**
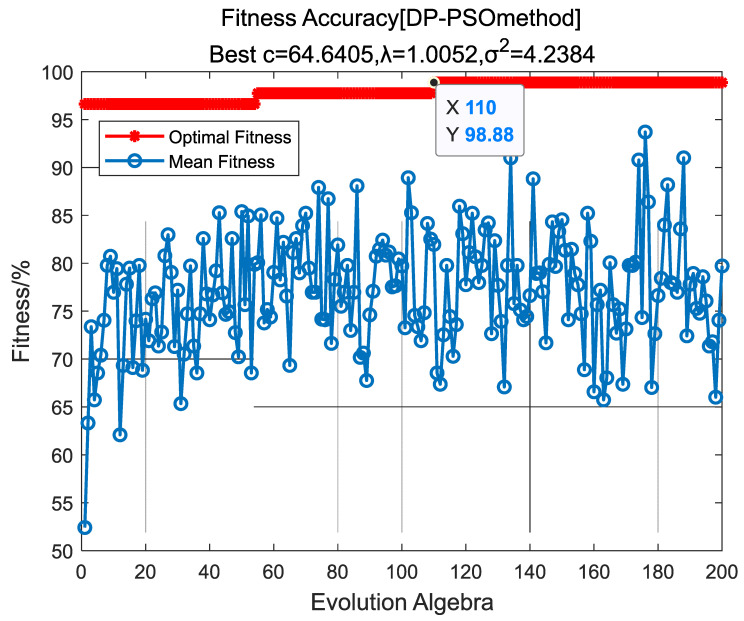
Fitness iteration curve.

The optimization results are as shown in Figure 5. The algorithm converged after 110 iterations, and the optimal parameter combination was obtained:

$$C=64.605,\;\lambda =1.0052,\;\sigma^{2}=4.2384.$$

### 3.4. Testing of Prediction-Model Performance

(1) Testing of the Prediction Efficiency of the Model

**Figure 6:**
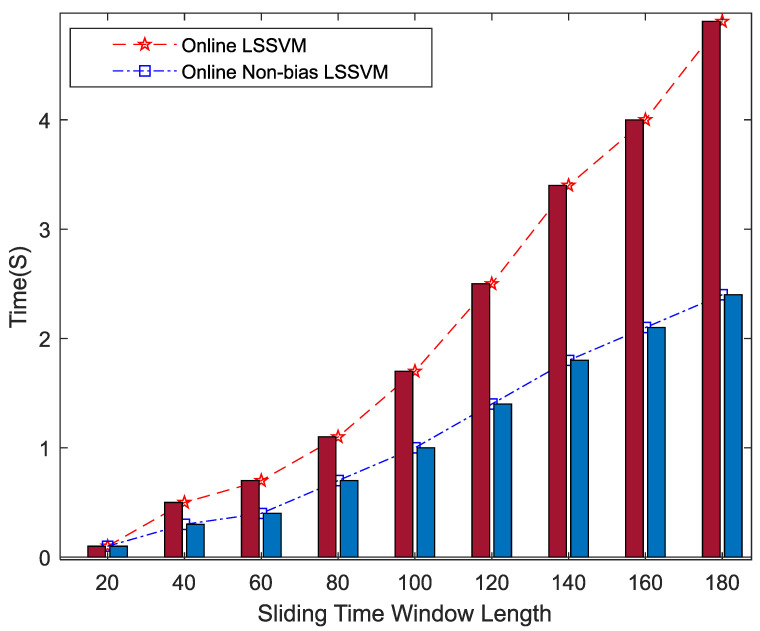
Running time of different time window lengths.

Output ripple voltages within 1–300 h were chosen as the training samples, and those within 301–625 h were used as the testing samples. The performance periods of 325 time-series predictions in nine groups were compared and analyzed under different lengths of sliding-time windows for the OLS-SVM and ONBLSSVM, whose results are shown in Figure 6. As Figure 6 shows, the ONBLSSVM has higher prediction efficiency than the OLS-SVM. With the increase in the sliding-window length, the prediction time of the ONBLSSVM increases more slowly than OLS-SVM, and this superiority becomes more significant as the sliding-time window becomes longer.

(2) Testing of Prediction Accuracy of the Model

**Figure 7:**
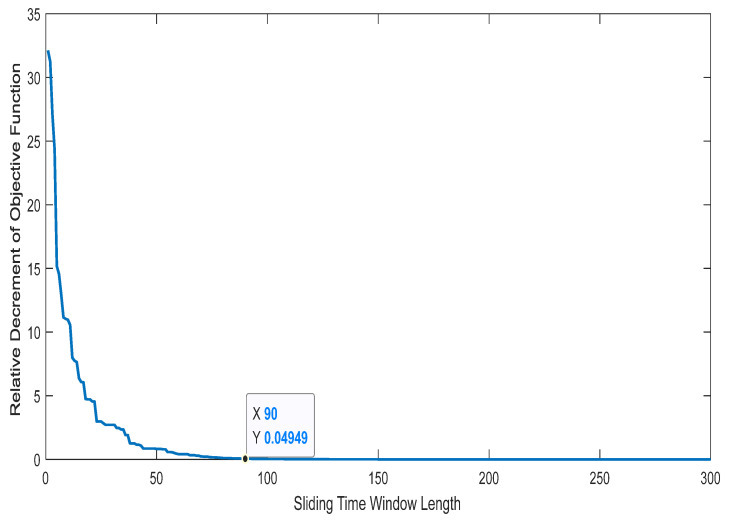
Adaptive selection of the length of the sliding-time window.

To balance the prediction accuracy and the modeling speed, it is necessary to choose a sliding-time window with appropriate length. The length can be calculated via the adaptive-adjustment algorithm (proposed in Section 2.3), and the simulation-experiment results are shown in Figure 7. With the prediction error θ≤0.01 V and the threshold ε = 0.05, the window length was finalized to be 90.

**Figure 8:**
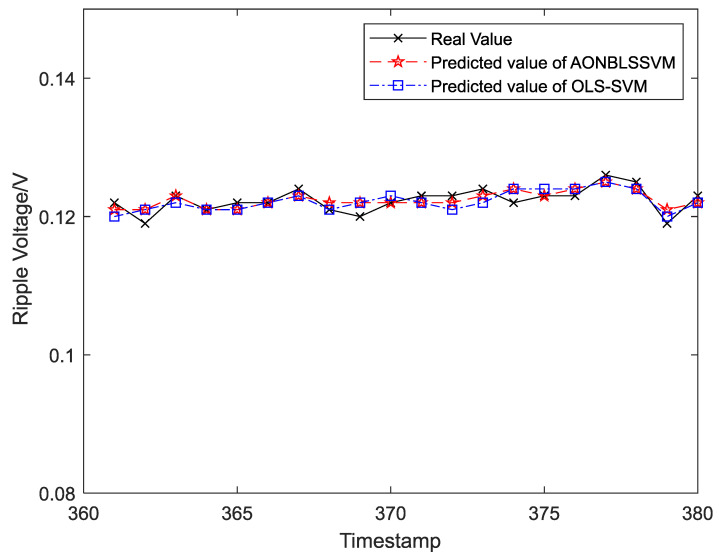
The prediction and fitting results of ripple voltage at time 361–380.

**Figure 9:**
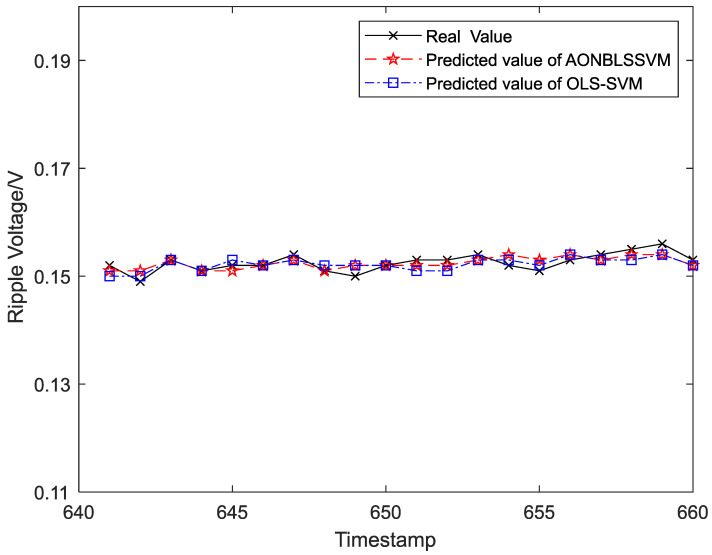
The prediction and fitting results of ripple voltage at time 641–660.

**Figure 10:**
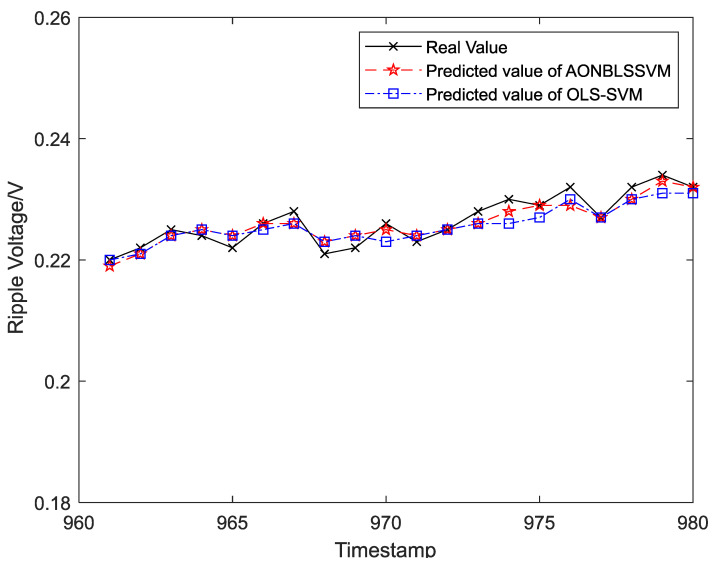
The prediction and fitting results of ripple voltage at time 961–980.

**Figure 11:**
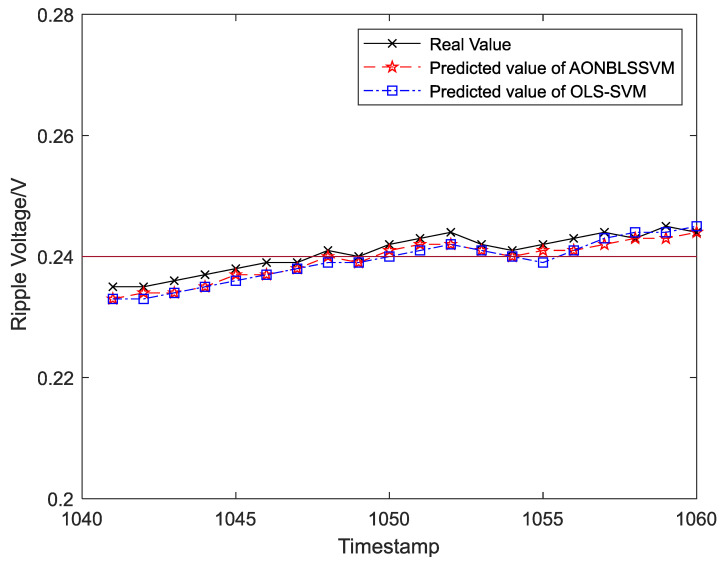
The prediction and fitting results of ripple voltage at time 1041–1060.

**Figure 12:**
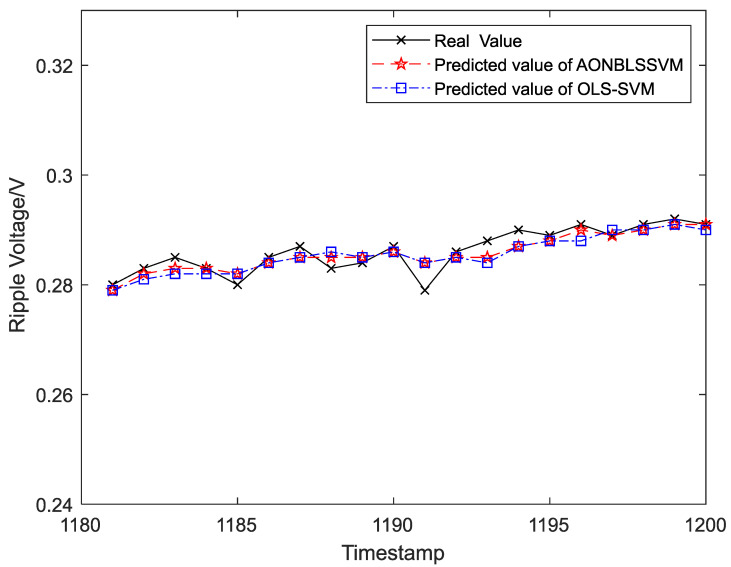
The prediction and fitting results of ripple voltage at time 1181–1200.

Output ripple voltages within 1–300 h were chosen as the training samples to form the sliding-time window and obtain the initial prediction model. To display the prediction effects more clearly and intuitively, output ripple voltages within 301–1400 h were classified into 55 groups in the time sequence, and each group was assigned 20 ripple-voltage values. Five data groups (100 ripple-voltage values in total) were chosen as test samples in time sequence for the evaluation of fitting between the real and predicted values. The curve-fitting results are shown in Figure 8, Figure 9, Figure 10, Figure 11 and Figure 12, the computational formulas for the chosen fitting-assessment indicator are as follows, where *n* is the number of test samples, $y_{i}$ is the real value, $\overset{\wedge }{y_{i}}$ is predicted value, and the specific computational values are provided in Table 2 and Table 3:

$$\mathrm{Mean}\;\mathrm{Average}\;\mathrm{Deviation}\;\left(\mathrm{MAD}\right)=\frac{1}{n}\overset{n}{\underset{i=1}{\sum }}\left|y_{i}-\overset{\wedge }{y_{i}}\right|$$

$$\mathrm{Mean}\;\mathrm{Average}\;\mathrm{Percentage}\;\mathrm{Error}\;\left(\mathrm{MAPE}\right)=\frac{100\%}{n}\overset{n}{\underset{i=1}{\sum }}\left|\frac{y_{i}-\overset{\wedge }{y}}{y_{i}}\right|$$

$$\mathrm{Theil}\text{’}s\;\mathrm{Inequality}\;\mathrm{Coefficient}\left(\mathrm{Theil}\;\mathrm{IC}\right)=\frac{\sqrt{\frac{1}{n}\sum_{i=1}^{n}{\left(y_{i}-\overset{\wedge }{y_{i}}\right)}^{2}}}{\sqrt{\frac{1}{n}\sum_{i=1}^{n}y_{i}^{2}}+\sqrt{\frac{1}{n}\sum_{i=1}^{n}{\overset{\wedge }{y_{i}}}^{2}}}$$

### 3.5. Analysis of Simulation Results

It can be seen from Figure 6 that the ONBLSSVM outperforms the OLS-SVM in terms of prediction efficiency, and the superiority becomes more significant when the sliding-time window is longer. Figure 11 shows that in combination with the output-ripple-voltage threshold (0.24 V), the DC–DC converter reaches its service life when the predicted value reaches the preset failure threshold (1048 h) for the first time. The fitting results of the predicted- and target-value curves in Figure 8, Figure 9, Figure 10, Figure 11 and Figure 12 vividly present that although the predicted values from the OLS-SVM are closer to real characteristic values than those from algorithm proposed herein at certain moments (379, 641, 975, 1057, etc.), our predicted-value curves generally fit better with the actual characteristic-value curves, suggesting that our algorithm has a higher prediction accuracy than the OLS-SVM.

The indicator data in Table 2 and Table 3 show that by comparing the three prediction and assessment indicators MAD, MAPE, and Theil IC in the five simulation experiments, our algorithm behaves better than the OLS-SVM, reconfirming its superior prediction accuracy.

## 4. Conclusions

In the AONBLSSVM algorithm, the bias term in the regression function was eliminated by optimizing the structural-risk forms of the LSSVM, and an online-learning method based on square-root decomposition was thus designed, which simplified the computation of the Lagrange multiplier and bias *b* during the dynamic updates of the model, avoided cumbersome computation, and reduced the modeling time. The adaptive selection of the sliding-time-window length was also realized to ensure the model could eliminate the constraints of old samples after adding new ones and achieve rapid updates. By adopting this method, monitoring data can be gradually injected into training sets over time, and historical training results can be exploited to the fullest in order to update the model online, thus effectuating the online monitoring of the DC–DC-converter circuit (a nonlinear time-varying system).

The AONBLSSVM algorithm relies much on the model parameters in terms of prediction accuracy. When the parameters are not well-configured, the prediction accuracy will be low. In DP-PSO, the concept of population co-evolution is introduced to the PSO to adjust the search strategies in a real-time manner so that the improved algorithm has stronger convergence and higher accuracy, thereby providing better prediction effects for the optimization of model parameters. DP-PSO is introduced for the optimized computation of model parameters, ensuring that a prediction model with higher accuracy will be established in a shorter time.

According to the simulation results, the circuit-fault-prediction model proposed herein showed good prediction and tracking capabilities for the soft fault of the DC–DC-converter circuit in a precise plot-seeder electric-drive system, and can be used for predicting the faults at the next moment in a fast and accurate manner. Furthermore, in combination with the circuit-failure threshold, it can provide a theoretical basis and support for predicting the service life of the DC–DC-converter circuit.

## Figures and Tables

**Table 1:** Parameters of DC–DC circuits within 0–15∆*t*.

Time	*ESR/*Ω	*C/*uF	*RON/*Ω	*RD/*Ω	*L/u*H	*UPP/*V
0	0.0200	1000.0000	0.0200	0.0100	33.00	0.092
1∆*t*	0.0209	997.7493	0.0206	0.0101	32.56	0.098
2∆*t*	0.0219	994.9920	0.0213	0.0103	32.12	0.106
3∆*t*	0.0230	991.6141	0.0222	0.0105	31.68	0.112
4∆*t*	0.0243	987.4759	0.0233	0.0108	31.24	0.120
5∆*t*	0.0257	982.4064	0.0246	0.0112	30.80	0.138
6∆*t*	0.0272	976.1958	0.0261	0.0118	30.36	0.147
7∆*t*	0.0290	968.5874	0.0280	0.0126	29.92	0.161
8∆*t*	0.0310	959.2666	0.0302	0.0139	29.48	0.173
9∆*t*	0.0333	947.8479	0.0329	0.0156	29.04	0.198
10∆*t*	0.0360	933.8591	0.0361	0.0180	28.60	0.236
11∆*t*	0.0390	916.7219	0.0400	0.0215	28.16	0.263
12∆*t*	0.0428	895.7276	0.0446	0.0264	27.72	0.291
13∆*t*	0.0473	870.0080	0.0502	0.0334	27.28	0.350
14∆*t*	0.0528	838.3950	0.0570	0.0433	26.84	0.433
15∆*t*	0.0600	799.8997	0.0065	0.0574	26.40	0.546

**Table 2:** Prediction-evaluation indexes of AONBLSSVM prediction model.

AONBLSSVM Prediction Model			
Experiment No.	MAD	MAPE (%)	Theil IC
1	0.95 × 10^−3^	7.796 × 10^−1^	4.747 × 10^−3^
2	1.00 × 10^−3^	6.561 × 10^−1^	4.017 × 10^−3^
3	1.20 × 10^−3^	5.300 × 10^−1^	3.278 × 10^−3^
4	1.30 × 10^−3^	5.410 × 10^−1^	3.219 × 10^−3^
5	1.45 × 10^−3^	5.088 × 10^−1^	3.248 × 10^−3^

**Table 3:** Prediction-evaluation indexes of OLS-SVM prediction model.

OLS-SVM Prediction Model			
Experiment No.	MAD	MAPE (%)	Theil IC
1	1.15 × 10^−3^	9.405 × 10^−1^	5.049 × 10^−3^
2	1.10 × 10^−3^	7.201 × 10^−1^	4.279 × 10^−3^
3	1.60 × 10^−3^	7.035 × 10^−1^	4.197 × 10^−3^
4	1.65 × 10^−3^	6.867 × 10^−1^	3.879 × 10^−3^
5	1.90 × 10^−3^	6.655 × 10^−1^	3.912 × 10^−3^

## Data Availability

Not applicable.

## Nomenclature

DC–DC	Direct Current to Direct Current
AONBLSSVM	Adaptive Online Non-bias Least-Square Support-Vector Machine
DP-PSO	Double-Population Particle-Swarm Optimization
OLS-SVM	Online Least-Square Support-Vector Machine
MOSFET	Metal-Oxide-Semiconductor Field-Effect Transistor
IGBT	Insulated Gate Bipolar Translator
*R*_*c*_	Equivalent-Series Resistance
$\bar{P_{c}}$	Average Power Loss of Capacitor
*I*_*C*_	Effective Value of Capacitive Current
*C_value_*	Capacity of Capacitor
SISO	Single Input–Single Output
MISO	Multiple Input–Single Output
CCM	Continuous Conduction Mode
DCM	Discontinuous Conduction Mode
*R_on_*	Drain-source On-resistance of Metal-Oxide-Semiconductor Field-Effect Transistor
SVM	Support-Vector Machine
LSSVM	Least-Square Support-Vector Machine
ONBLSSVM	Online Non-bias Least-Square Support-Vector Machine
*KKT* conditions	Karush–Kuhn–Tucker conditions
C	Penalty Factor
λ	Introduced Parameter
$\sigma^{2}$	Gaussian Kernel Function Breadth Factor
θ	Prediction-Error Threshold
ε	Refers to The Relative Decrement Threshold
*V* *o* *ut*	Output Voltage of Direct Current to Direct Current
*P* *o* *ut*	Output Power of Direct Current to Direct Current
*U* *pp*	Ripple Voltage
*t*	Time
∆*t*	Time Interval
MAD	Mean Average Deviation
MAPE	Mean Average Percentage Error
Theil IC	Theil’s Inequality Coefficient
